# Impaired gas exchange: accuracy of defining characteristics in children with acute respiratory infection^1^

**DOI:** 10.1590/0104-1169.0269.2581

**Published:** 2015-07-03

**Authors:** Lívia Maia Pascoal, Marcos Venícios de Oliveira Lopes, Daniel Bruno Resende Chaves, Beatriz Amorim Beltrão, Viviane Martins da Silva, Flávia Paula Magalhães Monteiro

**Affiliations:** 2 Doctoral student, Departamento de Enfermagem, Universidade Federal do Ceará, Fortaleza, CE, Brazil. Assistant Professor, Centro de Ciências Sociais, Saúde e Tecnologia, Universidade Federal do Maranhão, Imperatriz, MA, Brazil; 3 PhD, Associate Professor, Departamento de Enfermagem, Universidade Federal do Ceará, Fortaleza, CE, Brazil; 4 Doctoral student, Departamento de Enfermagem, Universidade Federal do Ceará, Fortaleza, CE, Brazil. RN, Prefeitura Municipal de Fortaleza, Fortaleza, CE, Brazil; 5 Doctoral student, Departamento de Enfermagem, Universidade Federal do Ceará, Fortaleza, CE, Brazil. Professor, Universidade Estadual do Ceará, Fortaleza, CE, Brazil; 6 PhD, Adjunct Professor, Departamento de Enfermagem, Universidade Federal do Ceará, Fortaleza, CE, Brazil; 7 PhD, Adjunct Professor, Curso de Enfermagem, Universidade da Integração Internacional da Lusofonia Afro-Brasileira, Redenção, CE, Brazil

**Keywords:** Nursing Assessment, Nursing Diagnosis, Signs and Symptoms, Respiratory, Child

## Abstract

**OBJECTIVE:**

to analyze the accuracy of the defining characteristics of the Impaired gas exchange nursing diagnosis in children with acute respiratory infection.

**METHOD:**

open prospective cohort study conducted with 136 children monitored for a consecutive period of at least six days and not more than ten days. An instrument based on the defining characteristics of the Impaired gas exchange diagnosis and on literature addressing pulmonary assessment was used to collect data. The accuracy means of all the defining characteristics under study were computed.

**RESULTS:**

the Impaired gas exchange diagnosis was present in 42.6% of the children in the first assessment. Hypoxemia was the characteristic that presented the best measures of accuracy. Abnormal breathing presented high sensitivity, while restlessness, cyanosis, and abnormal skin color showed high specificity. All the characteristics presented negative predictive values of 70% and cyanosis stood out by its high positive predictive value.

**CONCLUSION:**

hypoxemia was the defining characteristic that presented the best predictive ability to determine Impaired gas exchange. Studies of this nature enable nurses to minimize variability in clinical situations presented by the patient and to identify more precisely the nursing diagnosis that represents the patient's true clinical condition.

## Introduction

Nursing diagnoses related to respiratory function, specifically Impaired gas exchange, Ineffective airway clearance, and Ineffective breathing pattern have been frequently indicated in the literature as affecting people in different age ranges and situations^1^^-^ 6. Of these, Impaired gas exchange is a severe clinical condition defined as an "excess or deficit in oxygenation and/or carbon dioxide elimination at the alveolar-capillary membrane"^7^. 

According to the North American Nursing Diagnosis Association (NANDA-I), this diagnosis belongs to the domain Elimination and Exchange, Respiratory Function class, and the defining characteristics of it include: nasal flaring; headache upon awakening; cyanosis (in neonates only); confusion; abnormal skin color (e.g., pale, dusky); diaphoresis; decreased carbon dioxide; dyspnea; visual disturbances; abnormal arterial blood gases; hypercapnia; hypoxia; hypoxemia; restlessness; irritability; abnormal arterial pH; abnormal breathing (e.g., rate, rhythm, depth); somnolence; and tachycardia^7^. 

In acute respiratory infections, such as pneumonia, the functions of gas exchange in the lungs change according to the stages of the disease, resulting in two pulmonary changes: a reduced ratio between ventilation and perfusion and a decrease of the respiratory membrane's total surface area available. Both situations lead to hypoxemia and hypercapnia, which are defining characteristics of the Impaired gas exchange diagnosis^8^. Nonethless, even though conditions such as acute respiratory infection may lead to this nursing diagnosis, there are few studies addressing accuracy concerning this subject. 

Acute respiratory infections are most common during childhood and contribute to high levels of morbidity and mortality among children under the age of five. This is the most affected age range due to the susceptibility and immaturity of the respiratory tract at this age. Respiratory infections are classified as upper or lower respiratory tract, depending on its degree of involvement. Lower respiratory tract infections affect the lower airways and tend to last longer and, if not properly treated, may endanger the child's life^9^.

In the face of such a situation, nurses should carefully assess respiratory function to establish a nursing diagnosis regarding the patient's clinical condition early on and implement nursing interventions intended to reach its resolution. 

Establishing a nursing diagnosis, however, is a process full of uncertainties. For this reason, nurses should use a diagnostic rationale to find standard signs and symptoms compatible with the most likely diagnoses^10^. The identification of each new defining characteristic may confirm a diagnostic suspicion, eliminate one or redirect the nurse's attention to a human response, not considered to that point. Thus, studies of diagnostic tests can be used to determine the probability of the presence of a given nursing diagnosis^11^. 

Studies contributing to the establishment of useful defining characteristics help to minimize the variability existing in clinical situations presented by the patient and to identify the nursing diagnosis accurately that represents the true clinical condition. Usually, a single piece of clinical information is not sufficient to confirm the presence of a nursing diagnosis safely. Hence, a set of defining characteristics needs to be established and the relationship of these with plausible diagnostic hypotheses for a specific clinical situation needs to be verified^12^.

It is important to note that the prevalence and accuracy measures of the defining characteristics of a given nursing diagnosis vary according to the particularities of the population under study^2^^-^ 3 ^,^ 13 ^-^ 15. Additionally, the fact that the Impaired gas exchange diagnosis shares defining characteristics that are common to other nursing diagnoses may make its identification difficult. 

Given the previous discussion and aiming to improve nursing diagnoses' rationales, this study's objective was to analyze the accuracy of defining characteristics of the Impaired gas exchange diagnosis among children with acute respiratory infections.

## Method

Open prospective cohort study performed with a group of 136 children with acute respiratory infection for a consecutive period of at least six days and ten days at most to verify the occurrence of the Impaired gas exchange diagnosis. Prospective cohorts enable the complete and accurate measurement of information concerning clinical signs and symptoms considering temporal dependency among the variables. Due to the short duration of hospitalization of children with respiratory infections, we opted for an open cohort in which each individual was included at the time of admission. 

The study was conducted in two public children's hospitals located in the Northeast of Brazil. The study project was approved by the Institutional Review Board of one of the facilities. The parents or legal guardians were informed of the study's objectives and consented to data collection by signing free and informed consent forms.

Inclusion criteria were being admitted to the hospital for less than 48 hours and aged from zero to five years old. Acute respiratory infections included: pneumonia, bronchiolitis, sinusitis, pharyngitis and tonsillitis diagnosed by the facility's physician. Children who did not complete a minimum of six days of follow-up (discontinuity criterion) or had chronic diseases that changed the specific clinical condition of acute respiratory infection (e.g., congenital heart disease or cerebral palsy) were excluded from the study. 

The patients were recruited through consecutive sampling as they were admitted to the hospital and after verifying inclusion and exclusion criteria. The sample size was computed considering a confidence level of 95%, with a minimum sensitivity of 80%, with confidence intervals of 13%, and an estimated prevalence of 27.2%, according to a prior study^1^. Based on these values, an estimate of 134 children was found. The final sample was composed of 136 children who were assessed for a consecutive period of six to ten days, so that total number of assessments totaled 1,128. 

### Instrument for data collection

The instrument used to collect data was based on the defining characteristics of the Impaired gas exchange diagnosis according to the NANDA-I^7^ taxonomy and on literature addressing pulmonary assessment^16^^-^ 17. This instrument also included information related to the identification of children: sex, origin, medical diagnosis, number of hospitalizations, date of birth, and hospitalization. Operational definitions were created for each of the defining characteristics under study. 

Data were collected by previously trained members of a research group on nursing diagnoses. The training consisted of an eight-hour workshop so that the diagnostic methods inherent to the respiratory assessment were reviewed and standardized. Data were obtained through interviews and physical assessments were performed on inpatients in bed. 

### Diagnoses inference process 

The nurses selected to participate in the process of diagnostic inference belonged to the same research group. These nurses were initially trained to recognize the presence or absence of the Impaired gas exchange diagnosis based on the review of its defining characteristics. Afterwards, they were assessed in regard to their ability to correctly classify individuals with and without a diagnosis, based on their analysis of 12 fictitious clinical histories. The objective of this strategy was to enable all nurses to reach the same level of ability in inferring the diagnosis, so that more consistent and uniform assessments would be achieved^12^. Ten nurses divided into pairs participated in this stage. 

The total number of assessments (1,128) was divided into five blocks containing approximately 226 clinical histories each. The five blocks were assessed by different pairs to determine the presence or absence of Impaired gas exchange diagnosis. Each pair independently made a diagnostic inference of all the assessments concerning the same children. Inter-rater agreement measured using Kappa coefficient was 0.8948 (z=0.9605; p<0.001), which, according with the literature, is considered strong^18^. In the cases in which there was disagreement in regard to the diagnosis under study, the research team analyzed the assessments and established the presence or absence of the diagnosis.

### Data analysis 

Data were statistically analyzed using R software, version 2.12.1 (R Foundation for Statistical Computing, Vienna, Austria). The model of generalized estimating equation was adjusted to assess association between the defining characteristics and Impaired gas exchange. This method enabled analyzing all the assessments of the nursing diagnosis per person, taking into account the correlation between the repeated measures. The models of generalized estimating equations were based on a structure called the autoregressive model of order 1 (AR1), which assumes the presence of this diagnosis in the previous assessment^19^. The characteristics that represent association with the nursing diagnosis according to the model of the generalized estimating equations were assessed in regard to accuracy measures.

The analysis of the accuracy of defining characteristics was based on sensitivity, specificity, predictive values (positive and negative), likelihood ratio (positive and negative), and diagnostic odds ratio. The quality of the defining characteristics was assessed based on confidence intervals for positive and negative likelihood ratios. In this case, a defining characteristic is considered appropriate when the confidence intervals do not contain the value 1.

In this study, these measures are defined based on the descriptions presented in the literature^12^. Sensitivity represents the likelihood of a defining characteristic being present in patients with the nursing diagnosis under study. Specificity is the likelihood of a defining characteristic being absent in patients without the nursing diagnosis. A positive predictive value of a defining characteristic refers to the likelihood of the patient presenting the nursing diagnosis. If negative, the predictive value represents the likelihood of the nursing diagnosis being absent among patients without this defining characteristic.

## Results

The children assessed in this study were hospitalized for an average of 8.29 days (SD: ± 1.58); were 20.35 months old on average (SD: ± 3.11); and 58.1% were boys. The medical diagnosis most frequently observed was pneumonia (85.3%), though some children (11.8%) were admitted without having their respiratory infection specified and, in some cases, presented more than one medical diagnosis. 

**Figure 1.**
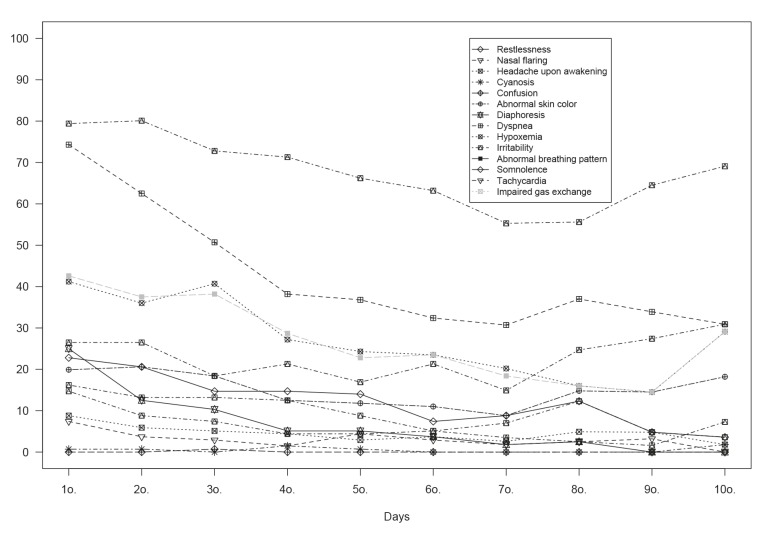
Distribution of defining characteristics of the diagnosis Impaired gas exchange during the monitoring of children with acute respiratory infection (n=136)

Figure 1 presents the distribution of the nursing diagnosis Impaired gas exchange and its defining characteristics during the time children with acute respiratory infection were monitored. The highest percentage (42.6%) of children with Impaired gas exchange was observed on the day one. On the days two and three, approximately 38% of children presented this diagnosis, while the number of children with this diagnosis kept decreasing up to the last day of monitoring. In regard to the defining characteristics, abnormal breathing was the most prevalent over the course of the ten days of assessment, the values of which ranged from 79.5% to 69.1% on the first and tenth days, respectively. Apnea was the second most frequent characteristic, presenting decreasing percentages over the course of the follow-up: 74.3% on the first day and 30.9% on the tenth day. Hypoxemia was frequently observed only in the first three assessments.

The model of the generalized estimating equation showed that the presence of the characteristics restlessness (OR = 11.37), cyanosis (OR = 87.83), abnormal skin color (OR = 10.06), hypoxemia (OR = 11642.1), and abnormal breathing (OR = 6.26) are associated with a greater likelihood of children with acute respiratory infection manifesting the Impaired gas exchange diagnosis when compared to children who did not present these characteristics (Table 1).

**Table 1.** Results of the model of the generalized estimating equation for all the assessments using Impaired gas exchange as the response variable (yes or no) and the defining characteristics as explanatory variables in the model (using AR1). Fortaleza, CE, Brazil, 2011

Defining characteristics	P-value	Odds ratio	CI 95%	
			Inf.	Sup.
Restlessness	0.029	11.373	1.285	100.64
Nasal flaring	0.116	3.771	0.721	19.727
Cyanosis	<0.001	87.836	11.614	664.28
Headache upon awakening	0.390	0.546	0.137	2.174
Confusion*	-	-	-	-
Abnormal skin color	<0.001	10.063	2.923	34.648
Diaphoresis	0.714	1.416	0.221	9.079
Dyspnea	0.875	0.931	0.380	2.278
Hypoxemia	<0.001	11642.1	2349.9	57677.3
Irritability	0.145	0.198	0.022	1.752
Abnormal breathing	0.001	6.269	2.172	18.092
Somnolence	0.235	2.154	0.607	7.641
Tachycardia	0.990	1.007	0.336	3.018

* There was no convergence for this model

The accuracy of the defining characteristics that presented statistical significance (p < 0.05) was assessed based on the results obtained through the model of the generalized estimating equation. The characteristic that presented the best measures of accuracy was hypoxemia (Sensitivity: 96.57%; Specificity: 98.38%; Predictive value+: 95.97%; Predictive value-: 93.21%). Other characteristics that also presented a high level of accuracy, above 70%, were: abnormal breathing (Sensitivity and Predictive value-); restlessness (Specificity and Predictive value-); cyanosis (Specificity, Predictive value+ and Predictive value-); and abnormal skin color (Specificity, Predictive value-). These results are presented in Table 2.

**Table 2.** Description of accuracy measures of the defining characteristics of Impaired gas exchange among children with acute respiratory infections. Fortaleza, CE, Brazil, 2011

Defining characteristics	Sensitivity	Specificity	Predictive value	
			Positive	Negative
Restlessness	20.81	89.33	43.79	73.85
Cyanosis	1.55	99.88	83.33	71.75
Abnormal skin color	17.70	86.10	33.73	72.37
Hypoxemia	96.57	98.38	95.97	93.21
Abnormal breathing	82.92	36.97	34.45	84.42

## Discussion

Usually, respiratory nursing diagnoses are a priority because they directly affect tissue oxygenation, which is a vital function. Identification of these diagnoses is particularly important among individuals with diseases in the respiratory tract due to impairment caused in their airways^20^. Among these diagnoses, Impaired gas exchange stands out because it is related to acute respiratory infection, which causes changes that negatively impact the functionality of the respiratory system, contributing to the onset of characteristic signs and symptoms. 

The prevalence of the diagnosis Impaired gas exchange (42.6%) verified in the first assessment diverged from another study^1^ that was also conducted with children with acute respiratory infections (27.2%). These divergent results may be related to the fact that that study was developed in a secondary public hospital, the children of which usually present more stable clinical conditions. This study, though, was conducted in a tertiary hospital, to which children with more severe conditions are referred, including those coming from the aforementioned secondary hospital. 

No studies addressing Impaired gas exchange using methodology similar to this study were found. There is, however, one meta-analysis^21^that addressed this diagnosis using data concerning the prevalence of defining characteristics presented in the literature to determine their accuracy measures. 

In this study, the characteristic hypoxemia presented the best measures of accuracy for the diagnosis Impaired gas exchange, which corroborates data from another study conducted with children with acute respiratory infections (Sensitivity: 90% and Specificity: 95%)^21^. This clinical indicator is associated with respiratory infections in children due to compromised respiratory function and alveolar ventilation. This occurs due to the retention of secretions, which may cause atelectasis, accruing from the occlusion of airways, compromising gas exchange and triggering hypoxemia^22^. 

Depending on the degree of hypoxemia, imbalance between the supply and demand of oxygen may lead to the appearance of the defining characteristic cyanosis, which, in this study, presented specificity and positive and negative predictive values. Nonetheless, no studies with statistically significant results were found for the purpose of comparison. 

In this study, abnormal respiration was a characteristic that presented high sensitivity (82.92%) and negative predictive value (84.42%) to determine Impaired gas exchange in children with acute respiratory infections. It is important, however, to highlight that the presence of this diagnosis was indirectly determined by the manifestation of at least one of the defining characteristics, namely: change in rhythm, respiratory rate or depth. 

The literature shows that increased respiratory frequency and depth can occur as the body's compensatory mechanism attempts to increase the flow of air in the respiratory system to fight high levels of carbon dioxide and hydrogen ions in the blood. These changes can be triggered by compromised airways due to the presence of secretions retained in the airways, which is common in children with respiratory infections^23^. 

Note that even though the defining characteristic abnormal breathing belongs to the Impaired gas exchange diagnosis, it is indirectly related to the defining characteristics changes in respiratory depth, tachypnea and bradypnea, which together constitute the Ineffective breathing pattern diagnosis^7^. Hence, it is possible that nurses have difficulty to precisely inferring respiratory nursing diagnoses when facing specific clinical situations. It is due to the fact that the process of inferring related nursing diagnoses may be affected when cases present similar defining characteristics or characteristics with descriptions that lead to the incorporation of information from another characteristic. 

This may explain the divergent results reported by a study conducted with adult patients with pulmonary diseases receiving mechanical ventilation and the results of another study addressing children with respiratory infections, since it did not present significant statistical values^21^.

Restlessness presented high specificity in the determination of Impaired gas exchange in the population under study, corroborating the results of another study conducted with a similar population, the specificity of which was 91%^21^. Restlessness may be triggered by conditions that change the respiratory state, such as acute respiratory infection, and is an important sign in cases in which there is hypoxic and respiratory failure. Inappropriate gas exchange intensifies signs of respiratory failure, making breathing a conscious effort, which results in apprehension, agitation and restlessness^8^.

Nonetheless, no statistically significant relationship was found for the restlessness characteristic in a study conducted with adult patients using invasive ventilation support^21^. It is known that patients using mechanical ventilation may be kept under sedation, which may compromise the identification of clinical manifestations of restlessness. Hence, we conjecture that this fact might explain the lack of statistical significance. 

Abnormal skin color (paleness) stood out due to specificity values and negative predictive value. Paleness may be related to the generalized mechanism of vasoconstriction as a consequence of neurogenic or hormonal stimuli and also due to decreased cardiac output, severe anemia, hypovolemia, acidosis or hyportermia^24^.

The relationship of the abnormal skin color characteristic with the Impaired gas exchange diagnosis may be explained by pathological processes such as pneumonia, which may obstruct the airways, trap gases, cause atelectasis, and increase dead space. Consequently, increase in the partial pressure of carbon dioxide in the blood leads to respiratory acidosis and stimulates chemo-sensitive bulb regions (central chemoreceptors), producing vasoconstriction and increasing peripheral resistance^8^.

In regard to the prevalence of the defining characteristics under study, dyspnea and abnormal breathing were the most frequently found. A possible explanation is that diseases affecting the exchange of respiratory gas through the pulmonary alveolar-capillary membrane, such as an acute respiratory infection, may promote disturbances in ventilation/perfusion, with an excess of carbon dioxide and an oxygen deficit. In this way, the body increases breathing as a compensating mechanism attempting to reach normal levels of these gases in the blood^8^.

The vigorous activity of the respiratory muscles contribute to the onset of abnormal breathing patterns that may manifest through dyspnea, changes in breathing frequency, rate or depth. These clinical indicators corroborate the results found in a similar study, in which these clinical manifestations were reported with high prevalence^1^.

Additionally, the result obtained by the Model of the generalized estimating equation for the Impaired gas exchange diagnosis showed that the presence of the characteristics of restlessness, cyanosis, abnormal skin color, hypoxemia, and abnormal breathing are related to an increased likelihood of this diagnosis occurring in children with acute respiratory infections. Respiratory and alveolar ventilation impairment caused by respiratory infections trigger adaptive compensatory mechanisms, which if not sufficient to stabilize breathing, may lead to other more severe manifestations. 

As previously discussed, hypoxemia caused by an imbalance between oxygen supply and demand may lead to abnormal breathing, restlessness, cyanosis, or paleness. Therefore, the set of these clinical signs may increase the likelihood that children with acute respiratory infection develop Impaired gas exchange. The close relationship of hypoxemia with this diagnosis was observed by the accuracy measures obtained. 

It is important to note that the scarcity of studies in the literature with a methodological design similar to this study limited the comparison of results. Therefore, we believe that similar studies addressing children with acute respiratory infections are needed to enable comparison with these findings. Note that the results presented here may have been influenced by the bias of incorporation and diagnostic assessment, which happens when prior knowledge concerning defining characteristics is incorporated during the process of diagnostic inference^25^. 

Despite the contribution of this study's findings concerning the accurate identification of the Impaired gas exchange diagnosis among children with acute respiratory infections, the results should be addressed with care, as most children were assessed in a hospital that provides care to patients with a greater probability of manifesting more severe clinical conditions. 

## Conclusion

The Impaired gas exchange nursing diagnosis was manifested in 42.5% of the sample. The most prevalent defining characteristics were abnormal breathing, dyspnea and hypoxemia. The model of generalized estimating equations showed that the concomitant presence of restlessness, cyanosis, abnormal skin color, hypoxemia and abnormal breathing are associated with an increased likelihood of the occurrence of this diagnosis in children with acute respiratory infections. 

In regard to accuracy measures, hypoxemia was the defining characteristic that predicted the occurrence of Impaired gas exchange diagnosis. Other characteristics, however, also presented high levels of sensitivity (abnormal breathing) and specificity (restlessness, cyanosis, and abnormal skin color).

It is believed that the determination of the predictive ability of these defining characteristics increases the reliability of the process of diagnostic inference and enables nurses to produce hypotheses consisting of more likely nursing diagnoses in the representation of the clinical situation presented by the patient.
